# Sizing femtogram amounts of dsDNA by single-molecule counting

**DOI:** 10.1093/nar/gkv904

**Published:** 2015-09-13

**Authors:** Dmitry Torchinsky, Yuval Ebenstein

**Affiliations:** Raymond and Beverly Sackler Faculty of Exact Sciences, School of Chemistry, Tel Aviv University, Tel Aviv 6997801, Israel

## Abstract

Modern molecular-biology applications raise renewed interest in sizing minute-amounts of DNA. In this work we utilize single-molecule imaging with *in situ* size calibration to accurately analyze the size and mass distribution of DNA samples. We exploit the correlation between DNA length and its fluorescence intensity after staining in order to assess the length of individual DNA fragments by fluorescence microscopy. Synthetic reference DNA standards are added to the investigated sample before staining and serve as internal size calibrators, supporting a robust assay for accurate DNA sizing. Our results demonstrate the ability to reconstruct the exact length distribution in a complex DNA sample by sizing a subset containing only femtogram amounts of DNA, thus, outperforming microfluidic gel electrophoresis which is the currently accepted gold standard. This assay may find useful applications for genetic analysis where the exact size distribution of DNA molecules is critical and the availability of genetic material is limited.

## INTRODUCTION

Sizing of DNA is one of the most fundamental practices in molecular biology. It is performed routinely to evaluate the outcome of polymerase chain reactions (PCR) (1), analyze restriction fragments (2), and assess the quality of extracted genomic or plasmid DNA (3). The growing utility of next generation sequencing (NGS), where the size distribution of DNA fragments is tightly controlled, raises renewed interest in sizing of ever smaller amounts of DNA (4).

The most widely used techniques for sizing DNA samples are gel electrophoresis (5) and its contemporary successors, such as pulsed field (6,7) and capillary gel electrophoresis (2,8–11). Of special relevance to DNA sequencing is a microfluidic based capillary gel electrophoresis device known as the 2100 Bioanalyzer, which has been commercialized by Agilent. This instrument is widely used for DNA sizing throughout NGS library preparation protocols and is considered the gold standard in sizing low amounts of DNA (12–15). All of the above techniques are based on partitioning a population of DNA molecules based on size and reading the average size distribution. They provide fairly accurate length and amount estimations, with the common setups being limited to nanogram amounts of DNA and capillary based techniques sensitive down to the picogram scale.

Another sizing approach is single-molecule flow cytometry (16–19). It is based on staining DNA with an intercalating dye (9,10,20), flowing individual DNA molecules through a confined laser spot and detecting their fluorescence intensity which later can be converted to length. The conversion is possible due to the fact that the amount of staining by intercalation is proportional to the length of the DNA molecule (21,22). Despite their single molecule sensitivity these techniques lack resolution and throughput and require complicated customized setups and regular calibrations (16,23).

Alternatively, single-molecule imaging by fluorescence microscopy allows visualizing individual DNA molecules immobilized on a microscope slide (11,24–27). Since visualization of DNA is often carried out by detecting fluorescence from an intercalating dye, it should in principle allow the sizing of these molecules based on their imaged intensity (28,29). Practically, since DNA staining is dependent on the exact experimental conditions and DNA to dye ratios, it is very hard to establish a constant, accurate conversion factor from intensity to length (30,31). Moreover, it has been reported previously that staining conditions affect the homogeneity of the sample resulting in poor separation and consequently in low accuracy and resolution of the measurements (32).

In this work we utilize single-molecule imaging with *in situ* size calibration to accurately analyze the size and mass distribution of a given sample. We use synthetic reference DNA standards as an internal size calibrator. The reference consists of two distinct DNA populations which are synthesized and covalently labeled with Cy5 by PCR (See Supporting Information). Spiking the investigated sample with the reference standard and staining the two together with an intercalating dye cancels out the factor of variable labeling conditions. Moreover, in order to be able to size samples of unknown concentration, we have adjusted the labeling conditions such that homogeneous staining is achieved at an excessive dye:bp labeling ratio. After imaging, the Cy5 labeling of the DNA reference molecules allows their separation from the investigated sample during data analysis. The intensities of the reference molecules are used to create a calibration curve for conversion of intensity to length, which allows sizing of the unknown DNA sample.

## MATERIALS AND METHODS

### DNA synthesis

All DNA standards were synthesized by PCR using the Eppendorf Mastercycler proS and Phusion High-Fidelity DNA Polymerase kit (NEB) with the provided GC buffer. The products were cleaned using QIAquick PCR Purification Kit (QIAGEN) with an adjusted protocol. For detailed PCR reaction protocols, thermocycling conditions, primer sequences and cleanup procedure modifications, see Supporting Information.

### DNA labeling with intercalating dye YOYO-1

A solution containing 1 ng of DNA suspended in TE buffer (10 mM Tris-Cl, 1mM EDTA, pH 8.5) was labeled with the intercalating dye YOYO-1 at dye:bp ratio of 1:1 (92.5 nM in 16 μl) and incubated overnight at 50°C. Before imaging, DTT was added to 0.2 M final concentration in order to protect the dye from photobleaching.

### Intercalated DNA sample deposition for imaging

For sample deposition, 8 μl of the sample containing 400 pg of DNA, were loaded on the contact interface between a positively activated glass coverslip and an untreated glass slide. The fluid was pulled in between the surfaces by capillary forces and DNA molecules were deposited on the positive coverslip surface. The glass coverslip activation is carried by incubating them overnight with a mixture of nitric and hydrochloric acids (200 ml and 100 ml, respectively). After acid incubation the coverslips are washed with deionized water and technical (96%) ethanol, blown dry with nitrogen and incubated overnight at 65°C in a mixture containing 600 μl of N-trimethoxysilylpropyl-N,N,N-trimethylammonium chloride, 50% in methanol and 200 μl of vinyltrimethoxysilane in 300 ml of deionized water. After silanization the coverslips were washed with deionized water and technical ethanol and stored in technical ethanol at 4°C.

### DNA Sample imaging, data acquisition and image analysis

The immobilized DNA molecules were imaged on an epi-fluorescence microscope (Till photonics, More) equipped with a high resolution EMCCD camera (Andor IXon888). A 150 W Xenon lamp was used for excitation with a filter set of 485/20ex and 525/30em (Semrock) for the YOYO-1 channel and a filter set of 650/13ex and 684/24em (Semrock) for the Cy5 channel. The data acquisition of multiple fields of view was carried out automatically using a motorized stage and an auto focus feature based on infrared reflection from the sample surface. Images were analyzed by software developed specifically for this application, resulting in statistics based on tens to hundreds of thousands of molecules per sample. The program detects individual DNA molecules and extracts their intensities after local background subtraction to account for uneven illumination of the field of view (See Supporting Information Figure S1, Figure S2 and Figure S3).

## RESULTS

**Figure 1.**
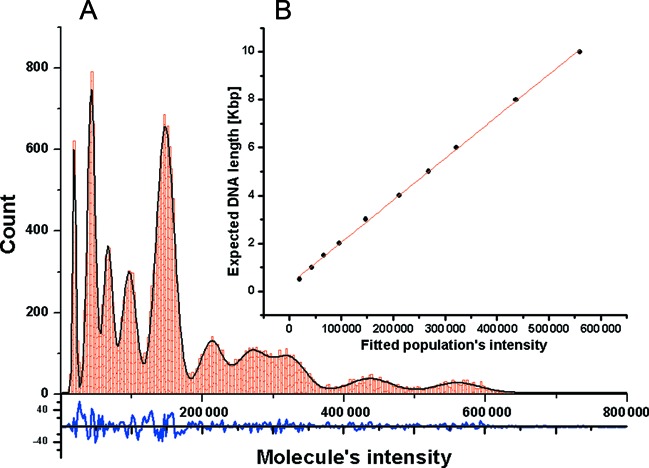
Single-molecule intensity distribution of a 1 kbp DNA ladder. (**A**) intensity distribution histogram of detected molecules (red), fitted Gaussians sum (black) and the residue of the fitting (blue). (**B**) a plot of expected DNA lengths in the ladder sample versus the centers of the fitted Gaussian peaks exhibits linear dependency with R^2^ = 0.99932.

In order to validate the linear dependency of intercalation intensity on DNA length, 1 ng of a 1 kbp DNA ladder (NEB) was labeled with the intercalating dye YOYO-1 and imaged. A histogram depicting the intensity distribution of the molecules in this DNA ladder sample is shown in (Figure 1A).

The resulting distribution contains 10 peaks corresponding to the 10 DNA populations in the sample. The data were fitted with 10 Gaussian functions (See Supporting Information) and the expected lengths of the DNA populations were plotted against the centers of the fitted peaks (Figure 1B). A linear fit to this plot validates the linear correlation between the imaged intensities and the expected molecular lengths of the various fragments in the DNA ladder, corresponding to previous observations (16).

In order to assess the dynamic range of such a detection scheme we also checked whether it is possible to detect samples containing shorter DNA fragments and confirmed that fragments down to 100 bp may still be separated with high resolution (See Supporting Information Figure S3 and Figure S4).

**Figure 2.**
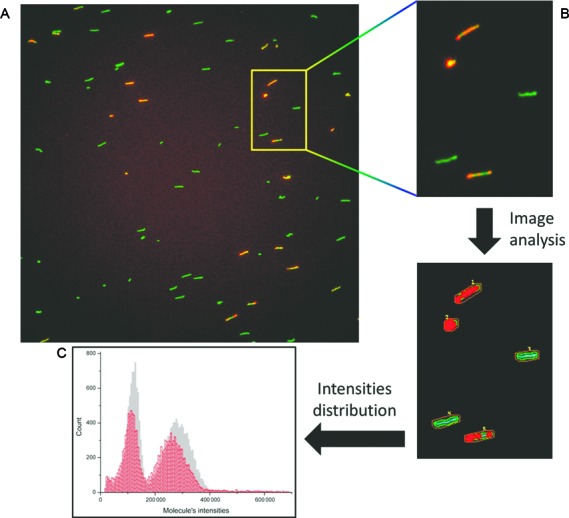
Analysis and calibration of labeled reference DNA sample. (**A**) representative raw fluorescence image of a single field of view (FOV) from a two color sample. YOYO-1 channel is green and Cy5 channel is red. (**B**) enlarged part of the FOV before (top) and after image analysis (bottom). The colocalized molecules are classified as reference and the rest as sample. (**C**) typical histogram of molecule intensities detected in a calibration sample. Grey data represent the intensities of the unlabeled molecules and the red data represent the intensities of the labeled ones.

Next, in order to facilitate quantitative sizing of an unknown sample, an internal size calibration standard was used. This reference standard is a DNA sample containing fragments of two known lengths, covalently labeled with multiple Cy5 fluorescent dye molecules. Two reference standards of 2991 bp and 7029 bp (referred to as 3 kbp and 7 kbp populations, respectively) were synthesized by PCR. The two calibration standards were imaged in order to verify the absence of non-specific PCR products and assure that all standard molecules are labeled with Cy5 (See Supporting Information Figure S5 and Figure S6). We then calibrated the reference standards in order to account for intensity changes caused by quenching of YOYO-1 fluorescence by Cy5 molecules (33). This was done by comparing the intensity distribution between the Cy5 labeled standards and unlabeled PCR products of identical lengths. The samples were labeled with YOYO-1 and imaged in two channels (YOYO-1 and Cy5). A raw composite of the two images is presented in Figure 2A were the YOYO-1 channel is green and the Cy5 channel is red.

The program used for data analysis locates molecules in both channels and detects labeled reference molecules based on their location overlap in the two color channels (Figure 2B). The colocalized molecules are classified as reference and the rest are considered to be sample molecules, resulting in two populations. The intensities of the detected molecules are plotted as a histogram showing the 3 kbp and 7 kbp populations, with grey data representing the unlabeled molecules and red data the Cy5 labeled reference standards, as shown in Figure 2C. Despite having identical lengths, the fluorescence intensity of the Cy5 reference standards is weaker than that of the unlabeled molecules of the same length, most likely due to energy transfer from YOYO-1 to Cy5 (33). Nevertheless, this comparison provides the effective apparent length of the reference standards, referred to as ‘imaged length’. Fitting the grey data with two Gaussian functions and plotting the theoretical length of the PCR products as function of the fitted centers, allows creating a calibration curve (See Supporting Information Figure S7). Fitting the red data with two Gaussian functions and placing their centers on the calibration curve, provides the imaged length of the labeled reference populations. Imaged lengths of 2721 ± 106 bp and 6456 ± 82 bp were measured respectively for our reference samples by averaging results from several samples. We note that this effect may most likely be minimized by using dye molecules with blue shifted emission relative to the intercalating dye.

**Figure 3.**
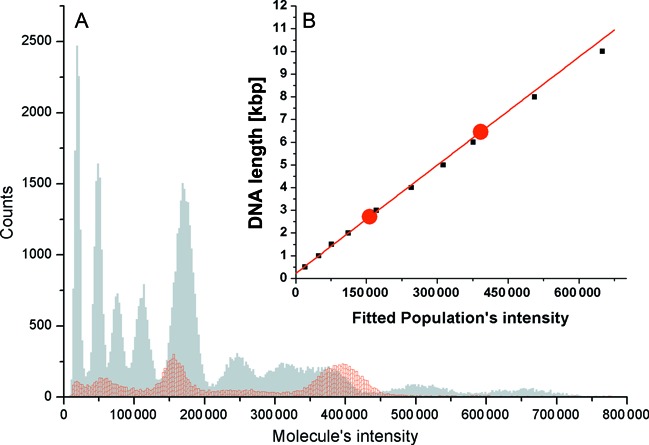
Molecule intensity distribution and calibration curve. (**A**) histogram of molecule intensities. Grey data represent the intensities of the sample molecules and red data represent the intensities of the reference molecules. (**B**) red circles represents the two calibration points and corresponding linear fit for conversion of molecule intensity to length in base-pairs. Black marks are the expected ladder lengths versus fitted intensities of the ladder populations. The experimental data are well represented by the calibration curve.

In order to simulate analysis of an unknown sample, 0.5 ng of 1 kbp DNA ladder (NEB) were mixed with 0.5 ng of reference sample, containing the previously calibrated 3 kb and 7 kb populations. The sample was labeled with YOYO-1 and imaged in two channels. After automatic analysis and classification of the imaged data, a histogram of sample and reference molecule intensities was plotted (Figure 3A).

**Figure 4.**
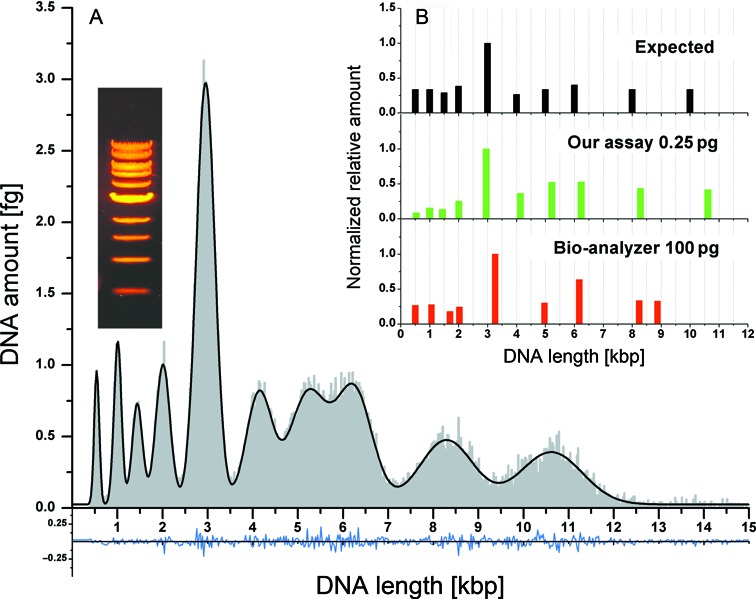
Experimental results. (**A**) histogram and fitted Gaussians sum of detected DNA amounts versus calculated DNA length. In grey are the experimental data, black curve represents the fitted Gaussians sum and blue is the fit residual. The insertion presents the DNA ladder as seen in conventional gel-electrophoresis setup. (**B**) graph of normalized relative amounts of the 10 populations in the sample. Black is the expected distribution according to the DNA ladder specifications sheet, green is our experimental distribution and red data are the results of the High Sensitivity assay of the Bioanalyzer microfluidic capillary gel electrophoresis system.

The reference data were fitted with two Gaussian functions and a calibration curve was constructed using the centers of the fitted peaks and previous calibration results (Figure 3B). The linear equation was used to convert the intensities of the sample molecules into base-pairs and a histogram of molecule lengths was created. The bin data of the histogram and molecular weight of DNA were used to convert the histogram counts into DNA amount in femtograms (Figure 4A).

A total of 250 fg of investigated DNA was sampled in this experiment and the length versus amount distribution was fitted with 10 Gaussian functions, where the center of each peak is the length of the population and its area is the amount of DNA for each population. The peak centers of the detected distribution closely match the expected length distribution (See Supporting Information Table S1). In fact, the size distribution is recovered even when the sampled portion of the data set is reduced down to around 10 fg of DNA (see Figures S8–S10 in Supporting Information).

We note that the distributions display a monotonous broadening of the fitted Gaussians with increasing DNA length (Figure S11 in Supporting Information). However, when calculating the relative estimation error (% error) we find that the error decreases as DNA length increases, corresponding to previously reported results (16) (Figure S12 in Supporting Information). Although the two-point calibration method used in our assay results in good size estimations and is practically easier to perform, we checked whether using additional calibration points may significantly improve the results. The data set was reanalyzed, taking some of the ladder populations as reference, and constructing three-point and four-point calibration curves (See Supporting Information Figure S13). The results show that the average estimation error of the assay stays practically the same (for both length and relative normalized amount), which indicates that two-point calibration, when performed accurately, represents a good compromise between performance and ease of use (see Supporting Information Table S1 and Table S2).

In order to benchmark our single-molecule assay, we compared our results to those obtained by microfluidic gel electrophoresis, a method considered the state of the art for such applications. In Figure 4B, the expected normalized relative amounts of the populations in the sample are presented in black and our experimental results are provided in green. The red data represent the results received for 100 pg of DNA using the high-sensitivity assay of the Bioanalyzer instrument (Agilent Technologies). We also ran samples of 500 pg and 1000 pg on the Bionalyzer in order to check its sizing capabilities for larger DNA amounts. The measured length and the relative normalized amount of each population for all the experiments are summarized in Tables S3 and S4 in the Supporting Information. It is interesting to note that the Bioanalyzer detected only 9 out of 10 populations for the 100 pg sample (missing the 4 kbp population). In fact, it also missed the same population for the 500 pg sample and managed to resolve it only for the most concentrated sample of 1000 pg.

We calculated the average total length estimation error for our assay to be 3.5% whereas that of the Bioanalyzer is almost twice as large for the 100 pg sample and increases with increasing sample concentration (See Supporting Information Table S3). In contrast, the Bioanalyzer was more accurate in reporting the relative amounts of DNA in each size population (See Supporting Information Table S4). We believe that this estimation error arises from the inhomogeneous distribution of the molecules on the glass coverslip and the fact that our assay only samples a subset of the entire sample as opposed to the Bioanalyzer which analyzes the sample as a whole. Future development that will allow confining the sample to the imaged region should overcome this limitation.

## DISCUSSION

In this work we have presented a single-molecule approach for quantifying the length and amount distribution of a DNA sample. The addition of a reference standard into the investigated sample prior to staining, cancels out labeling condition variations between samples whereas simultaneous imaging cancels acquisition parameters and imaging surface variations. The result is a robust and reproducible assay that is independent of exact labeling conditions and data acquisition method. The labeling ratios applied in this work show that homogenous intercalation can be achieved even with excessive labeling (1:1 dye:bp ratio) which is contrary to previous assumptions (32) and allows the application of this assay to samples of unknown concentration. We have demonstrated the ability to reproduce the size distribution of a complex DNA sample by imaging and analyzing a small portion of the sample down to low femtogram amounts. These low DNA amounts correspond to several percent of the total genomic DNA content in a single cell. Our results constitute a significant improvement over existing techniques for DNA sizing and may find useful applications for genetic analysis where the exact size distribution of DNA molecules is critical. Examples include characterizing free circulating DNA in plasma or urine (34–36), analyzing telomeres (37–45) and mitochondrial DNA (46,47), or in combination with sequencing library preparation. A clear advantage of the single-molecule approach is that it allows the study of samples where availability of genetic material is limited such as in medical micro-biopsies (48,49) or circulating tumor cells isolated from plasma (49,50). We hope that the high compatibility of this assay with automation and parallelization will allow the processing and analysis of multiple samples and may develop into a high-throughput platform for DNA analysis.

## Supplementary Material

SUPPLEMENTARY DATA
