# Supplementary material for: Sizing femtogram amounts of dsDNA by single-molecule counting
Source: Nucleic Acids Res. 2015 Sep 13;44(2):e17. doi: 10.1093/nar/gkv904 (PMC4737178; doi:10.1093/nar/gkv904)
Supplement: SUPPLEMENTARY DATA [file supp_44_2_e17__index.html]

Sizing femtogram amounts of dsDNA by single-molecule counting — Sizing femtogram amounts of dsDNA by single-molecule counting — SUPPLEMENTARY DATA 

# Sizing femtogram amounts of dsDNA by single-molecule counting

## SUPPLEMENTARY DATA

- SUPPLEMENTARY DATA
