# Supplementary material for: Sizing femtogram amounts of dsDNA by single-molecule counting
Source: Nucleic Acids Res. 2015 Sep 13;44(2):e17. doi: 10.1093/nar/gkv904 (PMC4737178; doi:10.1093/nar/gkv904)
Supplement: SUPPLEMENTARY DATA [file supp_gkv904_nar-01233-met-g-2015-File006.docx]

**Sizing femtogram amounts of dsDNA by single-molecule counting**

Dmitry Torchinsky^1^, Yuval Ebenstein^1,*^

^1^ Raymond and Beverly Sackler Faculty of Exact Sciences, School of Chemistry, Tel Aviv University, Tel Aviv, 6997801, Israel

* To whom correspondence should be addressed. Tel: +972-3- 6408698; Fax: +972-3- 6405794 ; Email: uv@post.tau.ac.il

**Supporting information:**

**Peak fitting procedure**

Gaussian fitting was carried out by Origin Pro software. The only parameters for the fitting were the initial guess of the peak centers provided by the user and the approximate average width of the expected peaks.

**Background illumination inhomogeneity**

The inhomogeneous excitation field may have an effect on the precision of the measurement. Although background signal is subtracted locally from each imaged DNA molecule, there is also an effect on the fluorescence intensity which is not taken into account in our analysis procedure. A representative image of the illumination profile is presented in Figure S1.


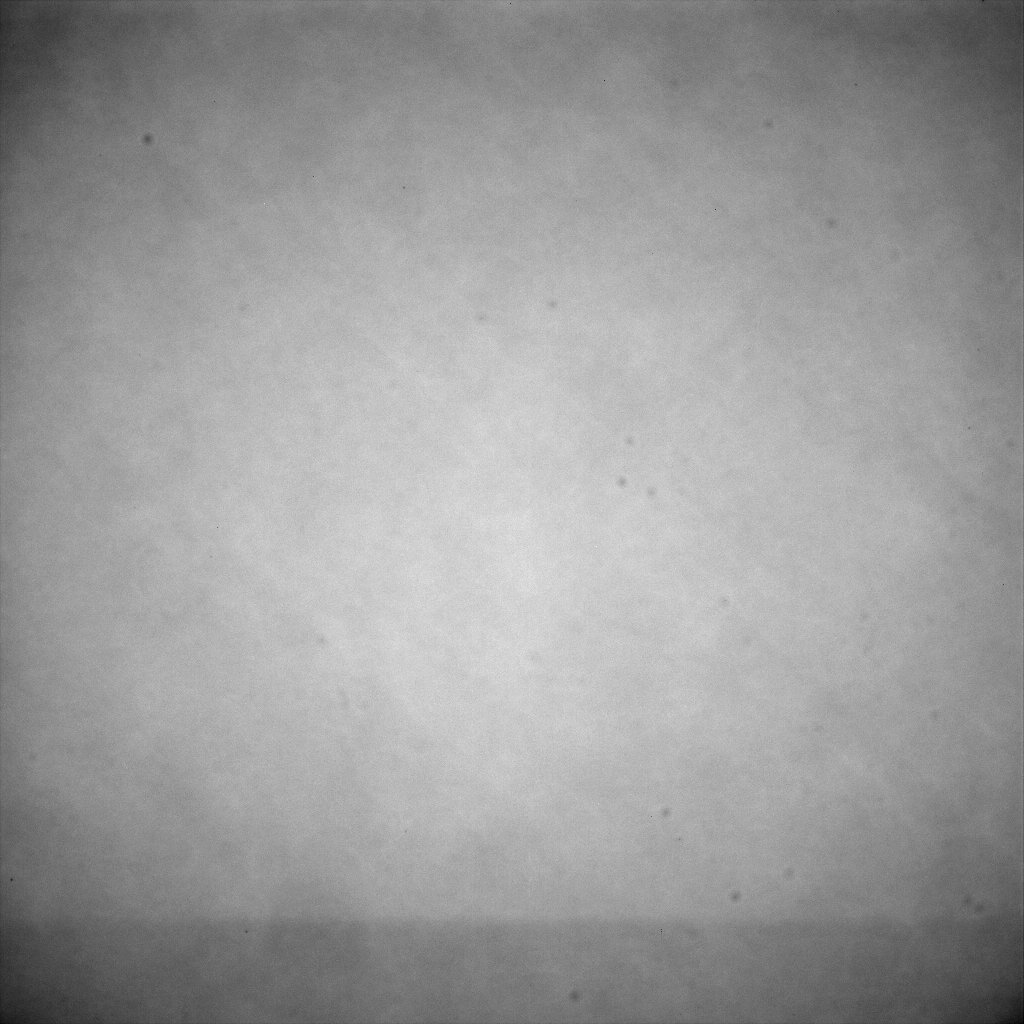


“**Figure S1.** Representative image of the background illumination profile, constructed from averaging all the images of an experimental data set”

The averaging procedure was carried out so that for each image all pixels above a certain value were discarded and their values substituted with the mean pixel value of the image after subtraction. In order to minimize the effect of inhomogeneous excitation on our results only molecules in the centeral 768x768 pixels in each FOV were analyzed. A vertical and horizontal line profiles were extracted from the image in Figure S1 and are presented in Figure S2.


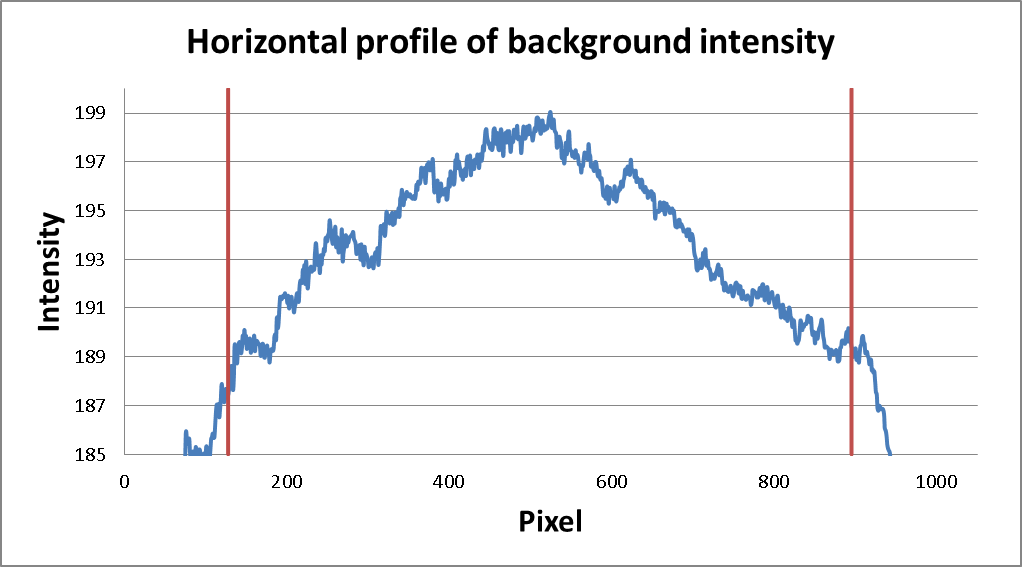

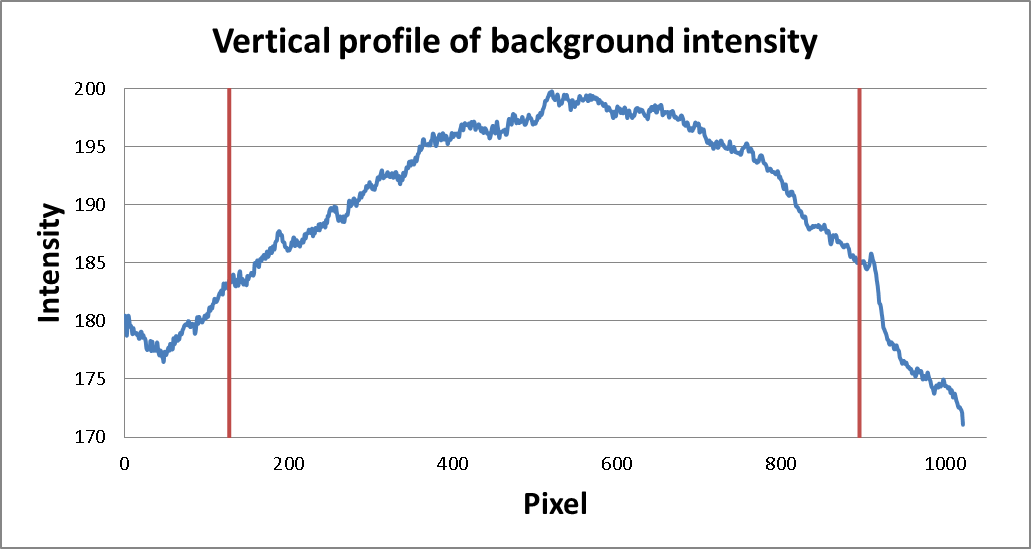


“**Figure S2.** Horizontal (left) and vertical (right) line profiles of the background illumination. The vertical lines on each graph indicate the analyzed part of an FOV for all the data presented throughout this work. The variation between the center and edges is less than 10% in both cases”

As presented in Figure S2, the center 768x768 of an FOV exhibits a variation of less than 10% percent. Nevertheless, we partially account for the uneven illumination by local background subtraction using an envelope around each molecule. Although this does not account for the uneven excitation it enhances the results as can be seen in Figure S3 by comparing the two peaks received for the same data set with and without our local background correction.


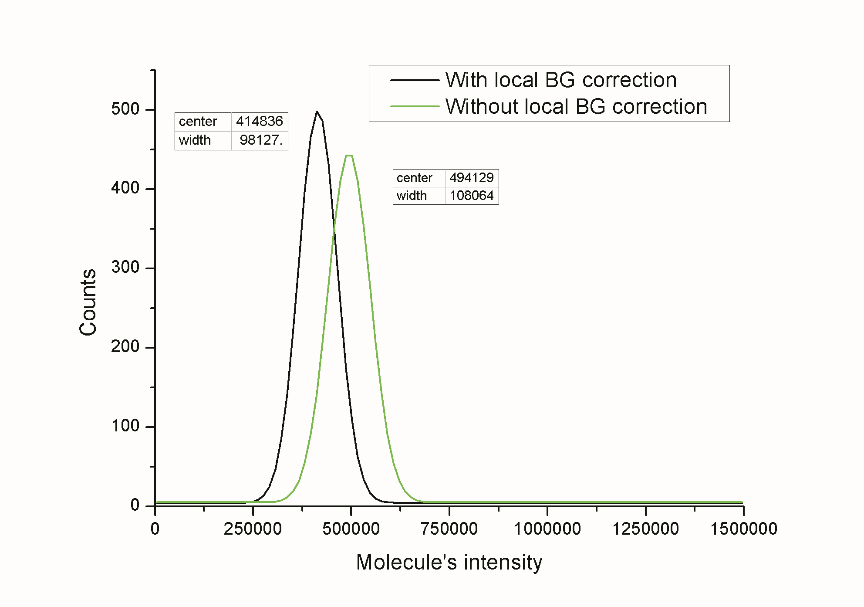


“**Figure S3.** Fitted Gaussian of intensity distribution of a single (100bp) DNA population with and without local background subtraction. Black data represents the corrected intensity whereas the green data represents the raw intensity. The width of the peak after BG correction is 98127 whereas before it was 108064 (arbitrary units).”

Figure S3 indicates that the uneven illumination causes a broadening of the peaks and that the local background subtraction partially accounts for this effect. In future work a blank image in the focus plane of the sample could be used to correct the uneven excitation by multiplying the intensity of each pixel of an image by a normalized factor deduced from the relative intensity of the same pixel in the blank image.

**Short DNA fragments sizing:**

In order to check the ability to size DNA samples containing shorter DNA fragments, we analyzed a DNA sample containing equal amounts of 200 and 400bp DNA molecules. Figure S4 represents the length distribution of the sample and the fitted Gaussians sum.


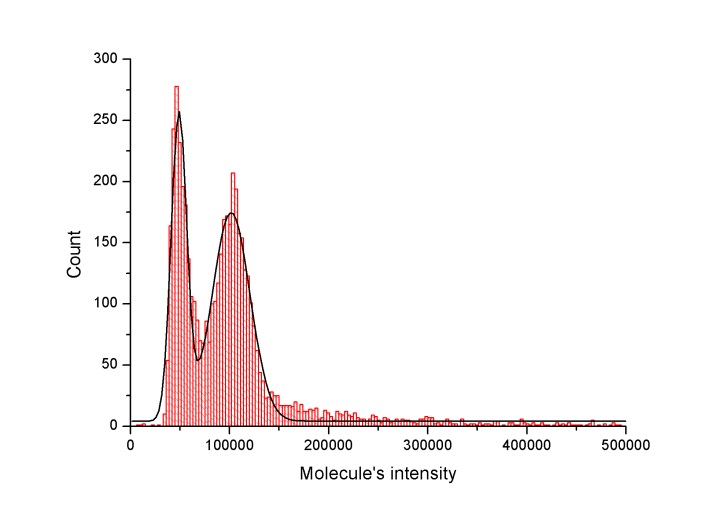


“**Figure S4.** Represents the amount vs. length distribution calculated for a 200 and 400 bp DNA sample. Black curve is the fitted Gaussian sum.”

Figure S4 shows that the two populations are fully separated which implies on future ability to size short DNA fragments with a reference DNA of an appropriate length.

**PCR protocols**

2991bp PCR no Cy5:

*Reaction conditions:*

Buffer: 5x Phusion GC (NEB) 30µL

Nucleotides: dNTP’s mix 10mM each (LAROVA GmbH), 3µL

Forward primer: 10µM (5’-ATTCGCCTGTCTCTGCCTAA-3’) (IDT) 3µL

Reverse primer: 10µM (5’-ACTTCCGGCGTGAATGTTAC-3’) (IDT) 3µL

Template DNA: Lambda phage DNA (NEB) 50ng/µL 3µL

Polymerase: Phusion High-Fidelity DNA polymerase (NEB) 1.5µL

DDW: 106.5µL

*Thermocycling conditions:*

Initial denaturation: 98^0^C, 300sec

30 Cycles: 98^0^C, 10sec 🡪 62^0^C, 30sec 🡪 72^0^C, 90sec

Final extension: 72^0^C, 600sec

*PCR purification:*

- Binding time of >15min
- Five washing steps of 700µLx1, 500µLx4
- Elution in 30µL warmed buffer (55^0^C) for >15min

7029bp PCR no Cy5:

*Reaction conditions:*

Buffer: 5x Phusion GC (NEB) 30µL

Nucleotides: dNTP’s mix 10mM each (LAROVA GmbH), 3µL

Forward primer: 10µM (5’-GCCACCTGTTACTGGTCGAT-3’) (IDT) 3µL

Reverse primer: 10µM (5’-GCGGCTACATGATACCCACT-3’) (IDT) 3µL

Template DNA: Lambda phage DNA (NEB) 50ng/µL 3µL

Polymerase: Phusion High-Fidelity DNA polymerase (NEB) 1.5µL

DDW: 106.5µL

*Thermocycling conditions:*

Initial denaturation: 98^0^C, 300sec

30 Cycles: 98^0^C, 10sec 🡪 65^0^C, 30sec 🡪 72^0^C, 210sec

Final extension: 72^0^C, 600sec

*PCR purification:*

As previously described.

2991bp PCR with Cy5:

*Reaction conditions:*

Buffer: 5x Phusion GC (NEB) 30µL

Nucleotides: dNTP’s mix 10mM each (LAROVA GmbH), 3µL

Labeled nucleotides: 5-Propargylamino-dCTP-Cy5 1mM (Jena Bioscience), 0.3µL

Forward primer: 10µM (5’-**/Cy5/**ATTCGCCTGT**/Cy5/**CTCTGCCTAA-3’) (IDT) 3µL

Reverse primer: 10µM (5’-**/Cy5/**ACTTCCGGCG**/Cy5/**TGAATGTTAC-3’) (IDT) 3µL

Template DNA: Lambda phage DNA (NEB) 50ng/µL 3µL

Polymerase: Phusion High-Fidelity DNA polymerase (NEB) 1.5µL

DDW: 106.2µL

*Thermocycling conditions:*

Initial denaturation: 98^0^C, 300sec

30 Cycles: 98^0^C, 10sec 🡪 62^0^C, 30sec 🡪 72^0^C, 90sec

Final extension: 72^0^C, 600sec

*PCR purification:*

As previously described.

7029bp PCR with Cy5:

*Reaction conditions:*

Buffer: 5x Phusion GC (NEB) 30µL

Nucleotides: dNTP’s mix 10mM each (LAROVA GmbH), 3µL

Labeled nucleotides: 5-Propargylamino-dCTP-Cy5 1mM (Jena Bioscience), 0.3µL

Forward primer: 10µM (5’-**/Cy5/**GCCACCTGTT**/Cy5/**ACTGGTCGAT-3’) (IDT) 3µL

Reverse primer: 10µM (5’-**/Cy5/**GCGGCTACAT**/Cy5/**GATACCCACT-3’) (IDT) 3µL

Template DNA: Lambda phage DNA (NEB) 50ng/µL 3µL

Polymerase: Phusion High-Fidelity DNA polymerase (NEB) 1.5µL

DDW: 106.2µL

*Thermocycling conditions:*

Initial denaturation: 98^0^C, 300sec

30 Cycles: 98^0^C, 10sec 🡪 65^0^C, 30sec 🡪 72^0^C, 210sec

Final extension: 72^0^C, 600sec

*PCR purification:*

As previously described.

**Reference labeling efficiencies and homogeneity**

1ng of synthesized DNA was labeled with YOYO-1 intercalating dye at dye:bp ratio of 1:1. The sample was incubated at 50^0^C overnight and then complemented with DTT to final concentration of 0.2M in a volume of 20µL. For sample deposition 8µL were loaded on the contact surface of a positively activated glass coverslip and an untreated glass slide. The imaging was done in two channels (YOYO-1 and Cy5) on an epi-fluorescence microscope (filter set - 485/20ex and 525/30em Semrock, 650/13ex and 684/24em Semrock). The acquired data was automatically analyzed by a custom program that extracted the molecules intensities and checked if the molecule is geometrically overlapping in the two channels. The detected molecules’ intensities were plotted on a histogram (Figure S5) to check the labeling homogeneity and the single peak in the distribution which indicates on a clean PCR product.


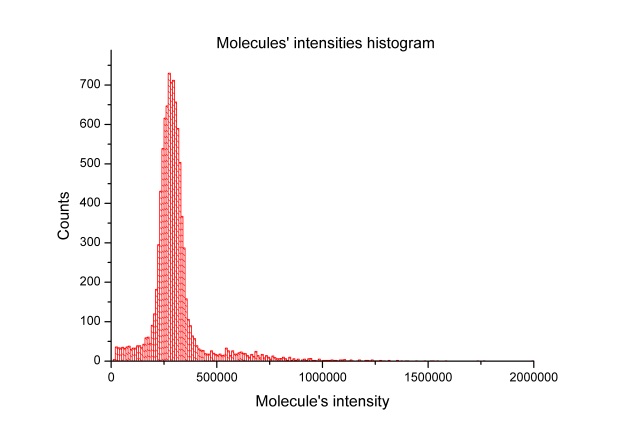

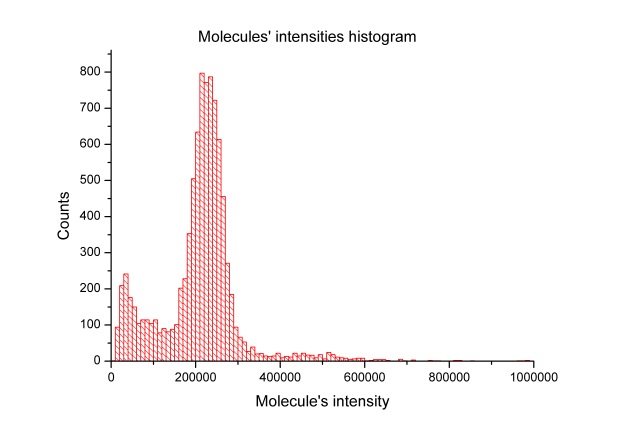


“**Figure S5**. Histogram of detected molecules’ intensities distribution for the 2991bp (left) and 7029bp (right) labeled PCR products. For the 3kbp product the distribution has a Gaussian profile which corresponds to theoretical expectations and indicates that the population has a single distinct length. The 7Kbp population has a Gaussian profile with a small population in the low intensities which most likely indicates that there is a small nonspecific product of the PCR reaction.”

In order to check how many of the detected molecules are labeled we look for spatial co-localization of the fluorescence signal in both channels (YOYO-1 & Cy5). High labeling efficiency is crucial in order to prevent the contribution of reference molecules to the investigated ones. Pie distributions depicting the number of co-localized molecules were constructed for each population (Figure S6).


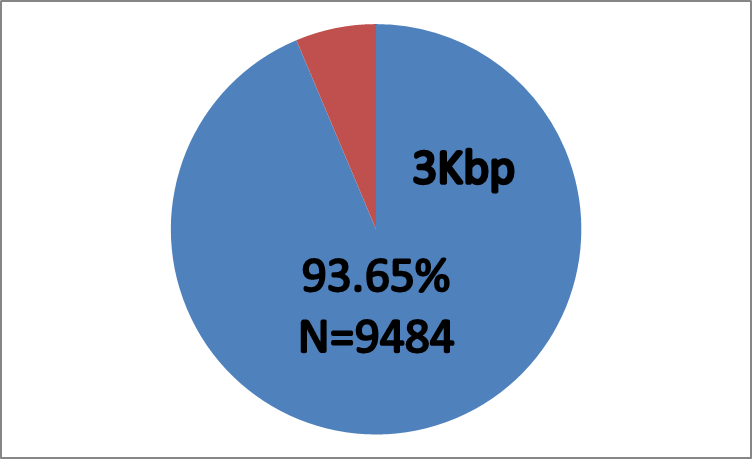

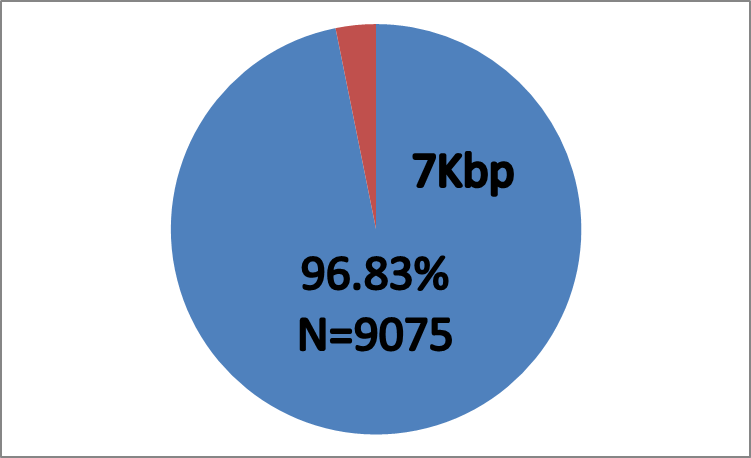


“**Figure S6.** Pie distribution of detected molecules’ co-localization status for the 2991bp (left) and 7029bp (right) labeled PCR products. The results shows that 93.65% of the detected molecules were labeled both with YOYO-1 and Cy-5 for the 3Kbp population and 96.83% for the 7Kbp.”

**Reference standard calibration:**

In order to account for the energy transfer between the YOYO-1 molecule and the Cy5 molecule the standard sample should be calibrated. The standard sample contains the 3kb and 7kb Cy5 labeled products, with the same number of copies from each population. To calibrate this sample, 4 portions were mixed with a similar sample containing 3kb and 7kb unlabeled PCR products and imaged as described above. The intensity distribution of the different populations is generally represented in Figure 2C, were the red data represents the labeled and the gray data the unlabeled molecules.

Figure 2C shows that the peaks of the labeled molecules are shifted to lower intensities due to the energy transfer and that there are less labeled molecules, presumably because they have lower adherence to the surface than the unlabeled ones. In order to account for this effect, the “imaged lengths” of the labeled populations were calculated for each sample. First the intensity distribution of the unlabeled molecules was fitted with two Gaussian peaks and the lengths of the populations were plotted as function of the centers of the peaks (Figure S7).


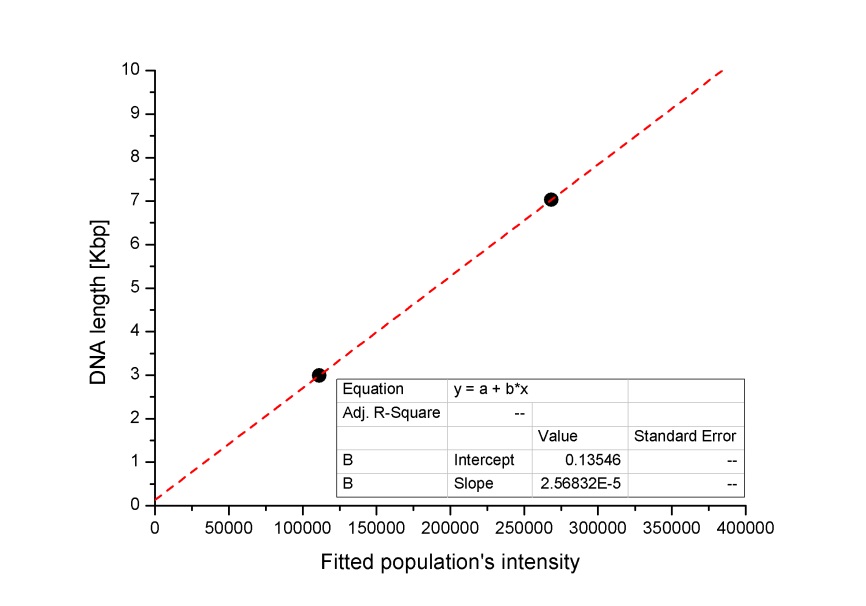


“**Figure S7.** A representative calibration curve of a standard sample. The theoretical lengths of the PCR products are plotted as function of the fitted peak’s centers of the unlabeled molecules’ distribution. The two points are fitted with a linear curve and the slope and intercept are recorded.”

To find the imaged length of each labeled population, the intensity distribution of the labeled molecules were fitted with two Gaussian functions and the centers of the peaks were substituted into the equation of the calibration curve. For each calibration sample the apparent “imaged length” was calculated and recorded. The mean imaged length for each labeled population was calculated (2.721Kbp and 6.456Kbp respectively) and used later on to create 2-point calibration curves for investigated samples.

**Assay detection limits:**

To check for the minimum amount of measured DNA that provides the correct length distribution of a sample, sub-sets of the whole dataset were analyzed. We randomly omitted images from the analysis reducing the amount of total sampled DNA for each analysis performed. We observe that down to 27fg of DNA still allows the Gaussian fitting algorithm to automatically detect the 10 populations in the sample. Furthermore, the resulting distribution is still highly representative even for 11fg of sampled DNA. The progressions in the shape of the distribution with decreasing sampling size are presented in figures S8-S10 depicting histograms for 124, 27, and 11 femtograms of DNA respectively.


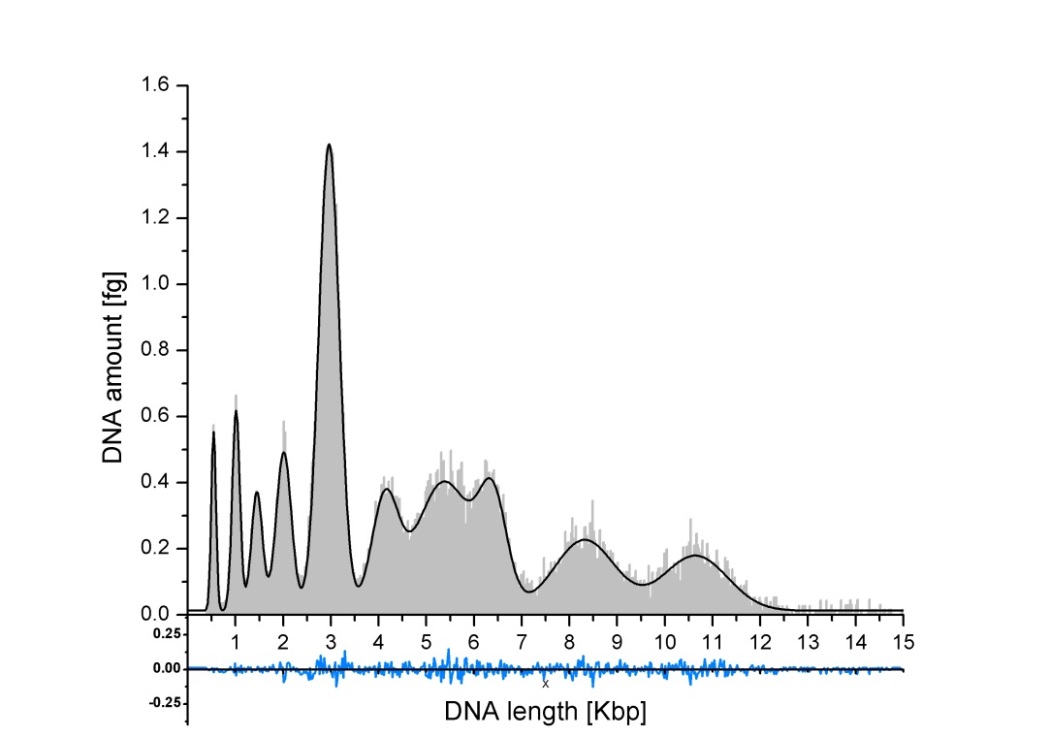


“**Figure S8.** Experimental results based on half of the total measured data set. In gray is the experimental data and the black curve represents the fitted Gaussians sum. The blue graph represents the fitting residual.”

Figure S8, shows that the noise in the histogram increased whereas the fitted lengths of the populations and the relative normalized amounts stayed almost the same as for the full data set. The total amount of DNA in this dataset is 123.6 femtograms.

The next step was to take a tenth of the entire measured population and to check whether it reproduces the results (Figure S9).


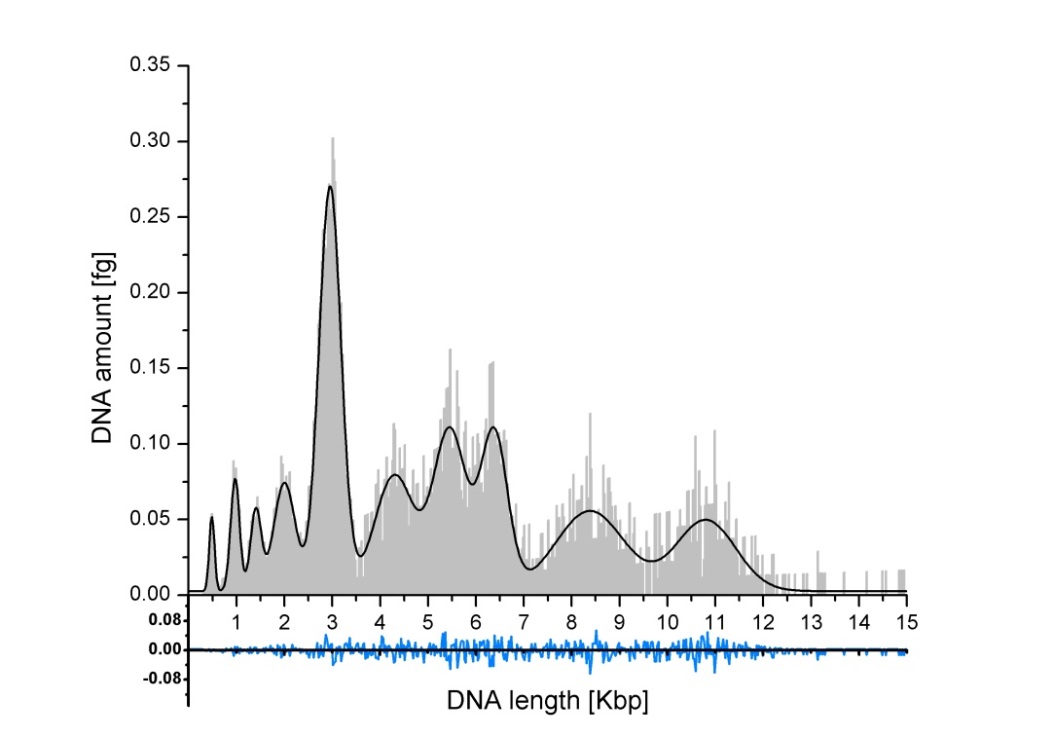


“**Figure S9.** Experimental results based on tenth of the measured data set. In gray is the experimental data and the black curve represents the fitted Gaussians sum. The blue graph represents the fitting residual.”

Figure S9 indicates that the noise of the histogram increased drastically but there are still 10 peaks in the distribution and the automatic Gaussian fitting algorithm still detects distinct populations. This result is based on sizing 27.5 femtograms of DNA. We further measured a population consisting of 11.3 femtograms of DNA as shown below (1:25 of the original data set).


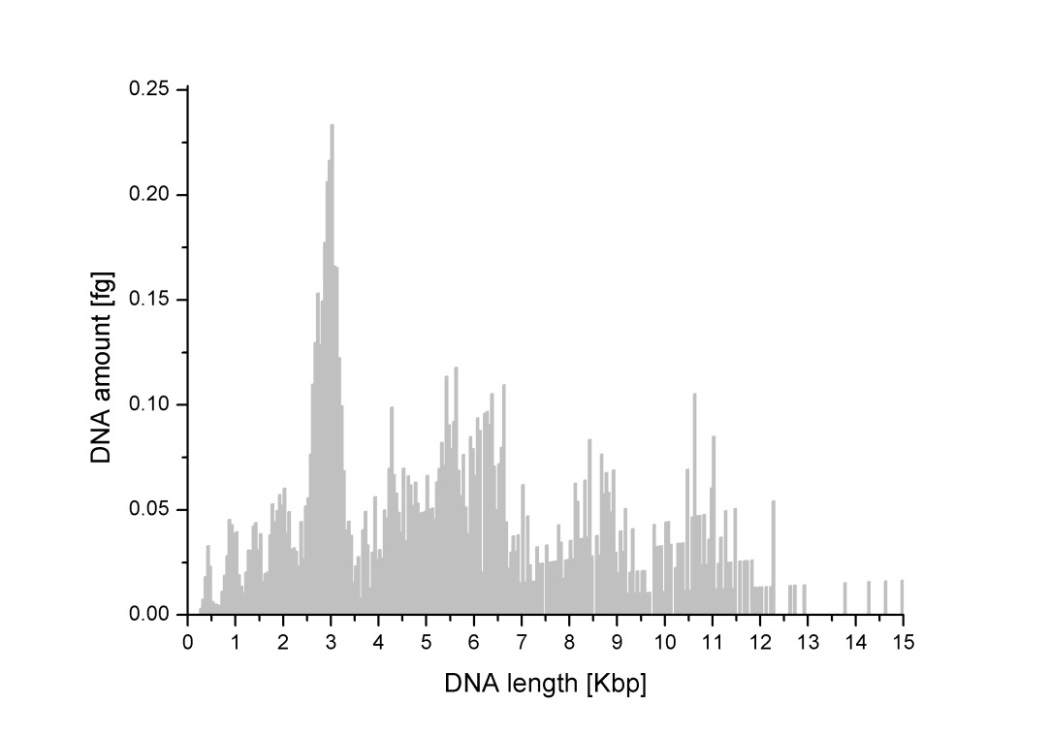


“**Figure S10.** Experimental results based on 1:25 of the measured data set.”

Figure S10 shows, that although the automatic Gaussian fitting algorithm did not recognize the peaks, the distribution is still highly representative.

**Assay estimation errors:**

The measured length of a population in a sample is deduced from the center of the fitted Gaussian peak, whereas the estimation error is reflected in the width of the fitted peak at half height. Our results show that, typically, the estimation error increases as the population lengthens (Figure S11) but the relative estimation error, which is the ratio between the center of a peak and its width at half height, decreases (Figure S12).


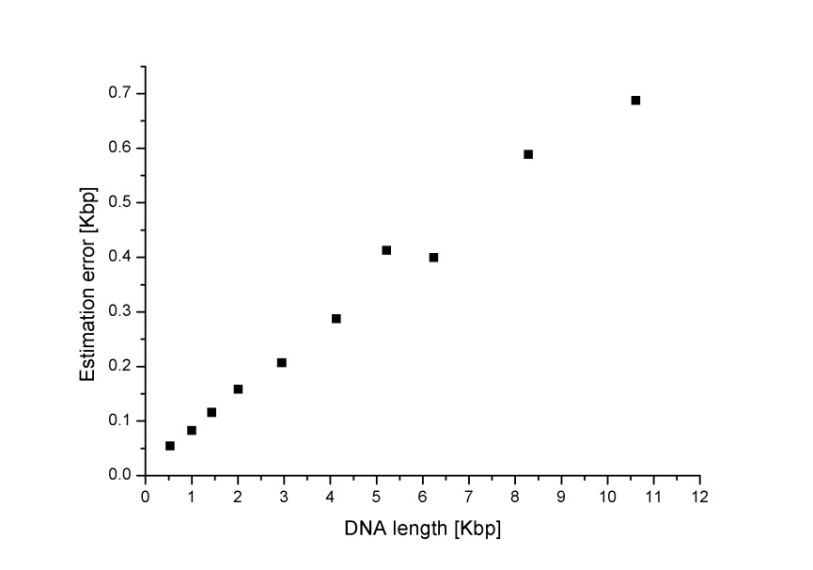


“**Figure S11.** Representative graph of estimation error dependency on measured DNA length for a 1kb DNA ladder. The graph shows that the estimation error increases with increasing population’s length.”


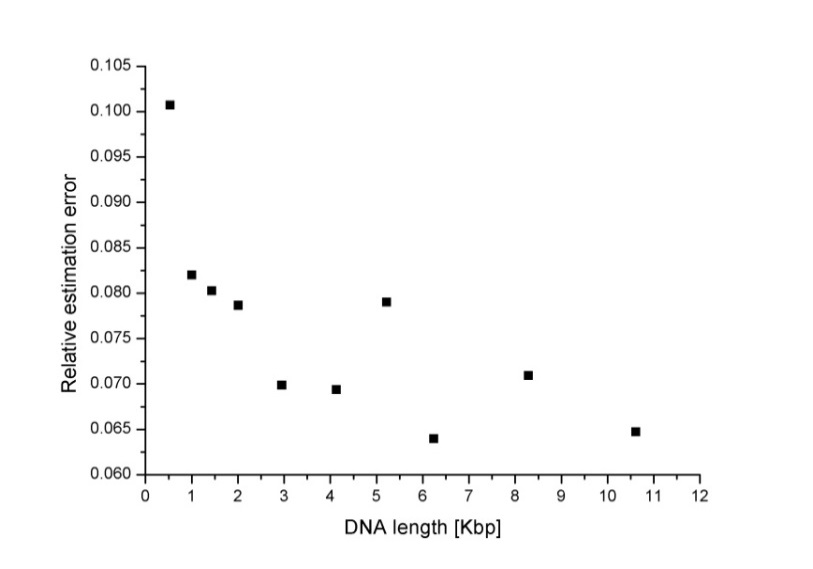


“**Figure S12.** Representative graph of relative estimation error dependency on measured DNA length for a 1kb DNA ladder. The graph shows that the estimation error decreases with increasing population’s length.”

**Multi-point calibration comparison**

To simulate whether the results of the assay improve with additional reference populations in the calibration sample, the data of the ladder sample was used. First of all, the intensity distribution of the ladder sample was fitted with 10 Gaussian functions. Then, the centers of the peaks corresponding to 0.5Kbp and 10Kbp were recorded. To simulate a three-point calibration curve the 0.5Kbp was added to the two reference populations and for a four-point calibration curve the 10Kbp was added as well. Figure S13 represents the three calibration curves received based on two, three and four points respectively.


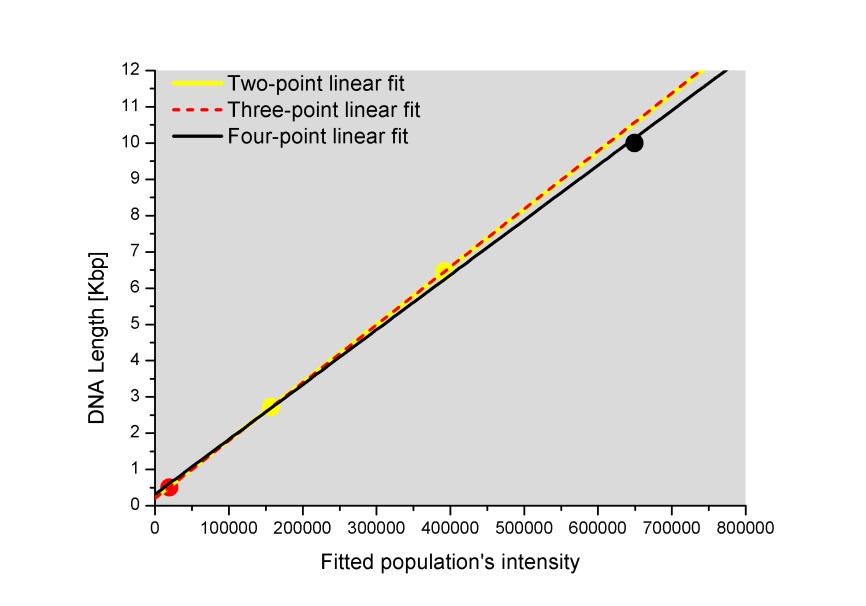


“**Figure S13.** Represents three calibration curves received with two, three and four points respectively. The two yellow points are the original reference populations whereas the red point is the edition of the 0.5Kbp population as the third calibration point and the black point is the edition of the 10Kbp population as a fourth calibration point”

Figure S13 shows that the received calibration curves are very similar and that the two and three point calibration curves practically overlap. Each calibration curve was used to size the ladder sample and the calculated lengths and their relative normalized amounts were recorded. To compare the results of the different calibrations, they were summarized in Tables S1 and S2.

“**Table S1.** Detected lengths comparison for two, three and four point calibration curves. For each estimated length the relative error from the expected length was calculated and presented on the right of the measurement. The bottom line represents the averaged relative error of each data set.”

“**Table S2.** Relative normalized amount comparison for two, three and four point calibration curves. For each population the relative error from the expected normalized amount was calculated and presented on the right of the measurement. The yellow line represents the population to which the rest were normalized to. The bottom line represents the averaged relative error of each data set.”

Table S1 indicates that there is not much difference between the results of the different calibrations, which indicates that the use of two calibration points is sufficient. Moreover, the relative normalized amounts stayed practically the same which also supports this statement (see Table S2). Based on these findings and accounting for practical consideration in preparing and using the reference standards, we conclude that the use of two points for further calibrations is acceptable.

**Comparison with Bioanalyzer results**

The detected lengths of the DNA ladder received by our technique and the Bioanalyzer in the HS assay are summarized in Table S3. Three different amounts of DNA were tested in the Bioanalyzer (100pg, 500pg and 1000pg). We also calculated the relative estimation error of each length from the expected one and it is provided in the adjacent column on the right of the respective assay. The bottom row shows the average value of the estimation error for each assay.

“**Table S3.** Detected length comparison of our experimental data and the Bioanalyzer results. Relative error from the expected length is presented to the right of each measurement. The bottom line represents the averaged relative error of each data set. Red represents populations that were not detected and the average error for those data sets was calculated based on the 9 detected populations.”

The results show that our assay is more precise than the Bioanalyzer under these conditions and that the bioanalyzer error increases with increasing amount of DNA. Moreover the Bioanalyzer failed to detect the 4Kbp population for both 100pg and 500pg samples. Only for the 1ng sample all ten populations were resolved but the average estimation error is more than threefold larger compared to that of our assay.

We further calculated the relative normalized amount of each DNA population and compared the results of each assay. The data is summarized in Table S4.

“**Table S4.** Relative normalized amount estimation comparison of our experimental data and the Bioanalyzer results. On the right of each measurement the relative error from the expected amount is presented. The bottom line represents the averaged relative error of each data set. Red represents populations that were not detected and yellow represents the population that the data was normalized to.”

The results show that the Bioanalyzer was more precise in its estimation and that its accuracy increases with increasing DNA amount.
